# Challenges to Comorbidity Management for Older HIV-Positive Individuals

**DOI:** 10.1093/geroni/igaa057.821

**Published:** 2020-12-16

**Authors:** Abigail Baim-Lance, Christine Kerr, Diane Addison

**Affiliations:** 1 Veterans Health Administration, Bronx, New York, United States; 2 Hudson River HealthCare, New Paltz, New York, United States; 3 CUNY Institute for Implementation Science in Population Health, New York, New York, United States

## Abstract

70% of individuals with HIV in the United States are 50 years or older. These individuals’ HIV is often well-managed, but they have acquired several comorbidities, including cardiovascular disease, non-AIDS defining cancers, renal disease, osteoporosis, liver disease, and neurocognitive disorders. Existing literature provides little guidance on structuring services to meet this burgeoning population’s complex clinical and aging-related needs. To inform an approach, between April 2015 and June 2018, we conducted 13 exploratory qualitative group discussions with patients, providers and administrators receiving or providing HIV services in New York’s Hudson Valley. We also conducted a retrospective electronic medical record chart review (n=50) of individuals >50 years receiving HIV care in 2017 at a Hudson Valley federally qualified health center (FQHC) to describe subspecialty referrals. Analysis of discussion groups highlighted challenges with initial access, the quality of encounters, and consistent follow-up. Patients thought ‘ageism’ contributed to poorer care quality, though extensive experience navigating the system was an advantage. The EMR review revealed patients receiving two referrals on average to (most commonly) ophthalmology/optometry (14%), gastroenterology (12%), dental (9%), cardiology (9%), and orthopedics (8%). Only half (54%) of scheduled referral appointments were attended. Documented barriers included insurance/costs, transportation, patient refusal, and fear of HIV disclosure. Findings suggest there are HIV-specific and non-specific barriers for older individuals with HIV, including perceived perceptions of aging. We have initiated a pilot study to investigate multilevel barriers and enablers to optimal comorbidity management. Gerontological insights brought to this analysis will support developing aging-appropriate clinical practices for this population.

